# 3D QSAR Pharmacophore Modeling, *in Silico* Screening, and Density Functional Theory (DFT) Approaches for Identification of Human Chymase Inhibitors

**DOI:** 10.3390/ijms12129236

**Published:** 2011-12-12

**Authors:** Mahreen Arooj, Sundarapandian Thangapandian, Shalini John, Swan Hwang, Jong Keun Park, Keun Woo Lee

**Affiliations:** 1 Division of Applied Life Science (BK21 Program), Systems and Synthetic Agrobiotech Center (SSAC), Plant Molecular Biology and Biotechnology Research Center (PMBBRC), Research Institute of Natural Science(RINS), Gyeongsang National University (GNU), 501 Jinju-daero, Gazwa-dong, Jinju 660-701, Korea; E-Mails: mahr@bio.gnu.ac.kr (M.A.); sunder@bio.gnu.ac.kr (S.T.); shalini@bio.gnu.ac.kr (S.J.); swan@bio.gnu.ac.kr (S.H.); 2 Department of Chemistry Education, Research Institute of Natural Science (RINS), Educational Research Institute, Gyeongsang National University, Jinju 660-701, Korea; E-Mail: mc7@gsnu.ac.kr

**Keywords:** chymase, pharmacophore, molecular docking, *in silico* screening, density functional theory, molecular electrostatic potential

## Abstract

Human chymase is a very important target for the treatment of cardiovascular diseases. Using a series of theoretical methods like pharmacophore modeling, database screening, molecular docking and Density Functional Theory (DFT) calculations, an investigation for identification of novel chymase inhibitors, and to specify the key factors crucial for the binding and interaction between chymase and inhibitors is performed. A highly correlating (*r* = 0.942) pharmacophore model (Hypo1) with two hydrogen bond acceptors, and three hydrophobic aromatic features is generated. After successfully validating “Hypo1”, it is further applied in database screening. Hit compounds are subjected to various drug-like filtrations and molecular docking studies. Finally, three structurally diverse compounds with high *GOLD* fitness scores and interactions with key active site amino acids are identified as potent chymase hits. Moreover, DFT study is performed which confirms very clear trends between electronic properties and inhibitory activity (IC_50_) data thus successfully validating “Hypo1” by DFT method. Therefore, this research exertion can be helpful in the development of new potent hits for chymase. In addition, the combinational use of docking, orbital energies and molecular electrostatic potential analysis is also demonstrated as a good endeavor to gain an insight into the interaction between chymase and inhibitors.

## 1. Introduction

**Figure 1:**
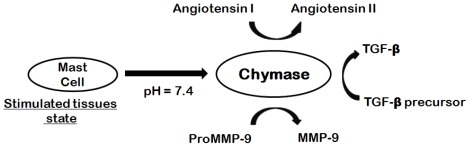
Chymase-dependent conversion of angiotensin I to angiotensin II and precursors of TGF-β and MMP-9 to their active forms.

Raised blood pressure, especially systolic pressure (hypertension), is one of the striking factors inducing various diseases like heart failure, stroke, myocardial infarction and arterial aneurysm, and is a leading cause of chronic kidney failure [1]. A treatment of hypertension is to decrease the circulating volume and/or to slack the blood vessels [2]. Angiotensin II has important roles not only in the regulation of blood pressure but also in the development of vascular wall remodeling [3]. Conversion of angiotensin I (Ang I) to angiotensin II (Ang II) is catalyzed by well-known angiotensin-converting enzyme (ACE), which is a metallo-proteinase with dipeptidyl-carboxypeptidase activity. However, chymase (EC 3.4.21.39) which is a chymotrypsin-like enzyme expressed in the secretory granule of mast cells, also catalyzes the production of angiotensin II in vascular tissues even when ACE is blocked (Figure 1).

Chymase converts Ang I to Ang II with greater efficiency and selectivity than ACE [4]. The rate of this conversion by chymase is approximately four fold higher than ACE. Chymase shows enzymatic activity immediately after its release into the interstitial tissues at pH 7.4 following various stimuli in tissues. Since chymase has no enzymatic activity in normal tissues, chymase inhibitors are expected to have high safety because chymase inhibitors may not have an effect on any other targets in normal tissues [5]. In order to generate Ang II, human, monkey, dog and hamster chymases cleave the angiotensin I at Phe8-His9 peptide bond. Chymase also converts precursors of transforming growth factor-β (TGF-β) and matrix metalloproteinase (MMP)-9 to their active forms thus contributing to vascular response to injury. Both TGF-β and MMP-9 are involved in tissue inflammation and fibrosis, resulting in organ damage [6]. Previous studies have demonstrated the involvement of chymase in the escalation of dermatitis and chronic inflammation pursuing cardiac and pulmonary fibrosis [7]. Therefore, inhibition of chymase is likely to divulge therapeutic ways for the treatment of cardiovascular diseases, allergic inflammation, and fibrotic disorders. Chymase inhibition may also be useful for preventing the progression of type 2 diabetes, along with the prevention of diabetic retinopathy [8]. Moreover, the role of chymase in inflammation has prompted its restorative value in diseases such as chronic obstructive pulmonary disease (COPD) and asthma [9].

Chymase inhibitors are imperative for elucidation of the physiological functions of chymase and potentially useful therapeutic agents. Several chymase inhibitors such as sulfonyl fluoride derivatives [10], Boc-Val-Pro-Phe-CO_2_Me [11], Z-Ile-Glu-Pro-Phe-CO_2_Me, (F)-Phe-COGlu-Asp-ArgOMe [12], *N*-(2-Naphthyl) carboxamido derivatives [13], *N*-(2,2-dimethyl-3-(*N*-(4-cyanobenzoyl)amino) nonanoyl)-l-phenylalanine ethyl ester [14], 3-benzylazetidine-2-one derivatives [15], 1,3-diazetidine-2,4-dione derivatives [16], methyllinderone derivatives [17], chloromethyl ketone derivatives [18], 1-oxacephem derivatives [19], and 3-(phenylsulfonyl)-1-phenylimidazolidine-2,4-dione derivatives [20] have been reported previously. In general, chymase inhibitors readily decompose in plasma, thus the stability of the chymase inhibitors in human plasma has always been a matter of great concern. For a drug candidate, it is essential to enhance the stability of the active compound in human plasma. So, there is always a dire need to search for more stable inhibitors with high activity against human chymase.

Many studies have indicated that computational approaches, such as predicting drug-target interaction networks [21], prediction of body fluids [22], predicting HIV cleavage sites in proteins [23,24], predicting protein metabolic stability [25], predicting signal peptides [26], identification of DNA Binding Proteins [27], predicting the network of substrate-enzyme-product triads [28], predicting protein subcellular locations [29,30], predicting proteases and their types [31], predicting antimicrobial peptides [32], predicting membrane proteins and their types [33], predicting GPCRs and their types [34], identifying nuclear receptor subfamilies [35], predicting gram-negative bacterial protein cellular locations [36], and predicting transcriptional activity of multiple site p53 mutants [37], can provide many useful insights and data for which it would be time-consuming and costly to obtain by experiments alone. Actually, these data, combined with the information derived from the structural bioinformatics tools (see, e.g., [38]), can timely provide very useful insights for both basic research and drug development. In view of this, the present study attempts to develop a new computational modeling method in the hopes it may become a useful tool for the drug development.

A quantitative structure-activity relationship (QSAR) study is a helpful approach to quantitatively understand the relationships between molecular structures of inhibitors and their biological activities [39–46]. Pharmacophore modeling and 3D-QSAR studies have been successfully applied previously for various drug discovery research, including glycoprotein (GP) IIb/IIIa antagonists, H_3_-antihistaminics, and dihydrofolate reductase inhibitors [47–51]. Electronic molecular features such as electron density, frontier molecular orbital density fields such as lowest unoccupied molecular orbital (LUMO), highest occupied molecular orbital (HOMO) and molecular electrostatic map have also been revealed to be significant in other QSAR studies to explain biological activity and molecular properties [52]. The HOMO density field was useful in a study of ACE inhibitors, and the LUMO density field was found to be important for explaining the TA100 mutagenicity [53,54]. Thus, determining molecular electronic properties responsible for the potent activity of selected chymase inhibitors should illuminate the fundamental molecular level forces responsible for their potency.

Various QSAR studies for chymase inhibitors have also been performed. The QSAR analysis of anhydride-type chymase inhibitors showed that aromatic substituents played an important role in determining the inhibitory potency of the compounds [55]. While, Hayashi and coworkers showed that introduction of various substituents in chloromethyl ketone derivatives resulted in a variation in their activity against human chymase [18]. A 3D QSAR model for the identification of stable chymase inhibitors has also been developed by Yuuki *et al*. 2003 [56].

**Figure 2:**
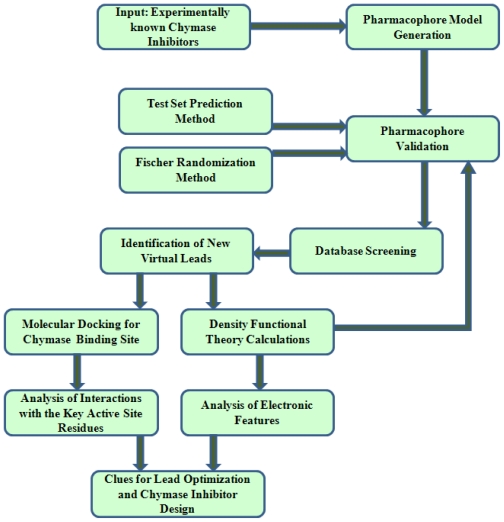
Flow chart elucidating the computational strategy applied in this study.

The subject of the present study is to develop QSAR models and explore the key molecular features of chymase inhibitors influencing the protein-ligand binding and interaction, by exploring the dependence of inhibitory activities upon various physiochemical properties of these compounds. In order to accomplish these tasks, an exclusive computational strategy is applied by using various QSAR model building techniques such as pharmacophore modeling, molecular docking, and Density Functional Theory (DFT) (Figure 2).

In the first phase of calculations, a pharmacophore model (Hypo1) comprising key chemical features for the identification of novel and diverse chymase inhibitors has been generated. After validation, this pharmacophore model is used as a 3D structural search query to find new classes of compounds with similar chemical features from chemical databases. The obtained hits are scrutinized based on their estimated activity and calculated drug-like properties. Molecular docking is also performed for the evaluation of compounds for important binding site interactions and affinity. Finally, we have carried out DFT-based QSAR studies on a set of chymase inhibitors retaining structural diversity and a wide biological activity range, along with potent hits retrieved by newly developed pharmacophore model (Hypo1). The objective of this DFT study is two-fold. One purpose is to derive the QSAR model itself and the other is to scrutinize the usefulness of conceptual DFT quantities. Moreover, it also served as a validation technique for the generated pharmacophore model. Various electronic properties such as LUMO, HOMO, and locations of molecular electrostatic potentials, are computed. The results of this study are expected to explore the crucial molecular features contributing to binding specificity and be useful for understanding the molecular mechanism by which these compounds act and can be further utilized to get compounds with better activity by rational modification.

## 2. Results and Discussion

### 2.1. Pharmacophore Modeling

**Figure 3:**
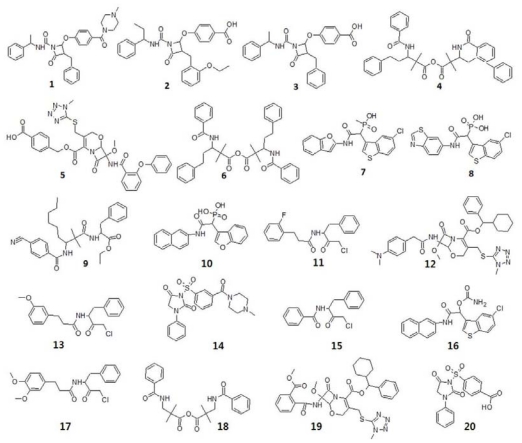
2D molecular structures of training set compounds.

One of the main objectives of the present study is to generate a pharmacophore model for the identification of novel chymase inhibitors. To accomplish this, ten hypotheses with imperative statistical parameters were generated by *HypoGen* module of DS using a training set of 20 compounds (Figure 3).

The hypotheses are generated with cost functions and correlation values by which they are estimated. The fixed cost, total cost and null cost values are calculated by *HypoGen* module during the hypotheses generation. The fixed cost is the lowest possible cost representing a hypothetically simplest model that fits all data perfectly, whereas the null cost value is equal to the maximum occurring error cost. For a more statistically significant hypothesis, there should be greater difference between these two cost values. The possibility of correlating the experimental and estimated activity data enhances to 75–90% with a cost difference of 40–60 bits between the total and null cost values [57,58]. In the present work, the null cost value of the top 10 hypotheses is 182.366 and the fixed cost value is 75.791. Thus, a difference of 106.575 bits between fixed cost and null cost consigns to a meaningful pharmacophore model. Moreover, the total cost of the generated hypothesis should be closer to the fixed cost. All ten generated hypotheses scored a total cost closer to the fixed cost which leads to a good model. Statistically significant factors which include cost values, correlation coefficients (*r*), pharmacophore features, and root mean square deviations (RMSDs) of all 10 hypotheses are listed in Table 1.

The configuration cost enumerates the entropy of the hypothetical space and its value should not exceed a maximum value of 17 for a significant pharmacophore model [59,60]. The configuration cost value of 16.601 was obtained for this pharmacophore generation calculation.

**Figure 4:**
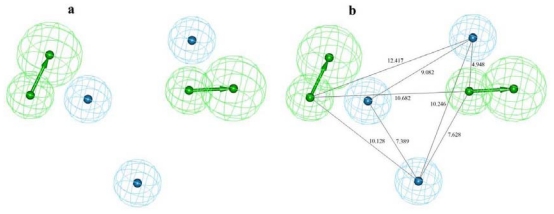
The pharmacophore model, Hypo1, showing two hydrogen-bond acceptors (HBAs) and three hydrophobic aromatic (HY-AR) features (**a**) and with distance constraints (**b**).

Seven of the 10 hypotheses were made of five pharmacophoric features while another three had shown four features. The HY-AR was the common feature among all hypotheses. Nine of the 10 hypotheses had Hydrogen-bond acceptor (HBA), three hypotheses had ring aromatic (RA) while only one hypothesis was made of hydrogen bond donor (HBD). Hypo1 consists of two HBA and three HY-AR features and scored the better correlation and cost difference values. The RMSD value indicates the quality of “prediction” for the training set. The RMSD of all ten hypotheses ranged from 1.176 to 1.421 Å while the Hypo1 showed the lowest RMSD value of 1.176 Å. The correlation coefficient for the Hypo1, 0.942, represents a good correlation by linear regression of the geometric fit index. All these results construe that Hypo1 is the best ranking pharmacophore model among other hypotheses (Figure 4).

On the basis of the activity, compounds belonging to training and test sets were categorized into activity scales: most active (++++, IC_50_ (inhibitory concentration) < 20 nM); moderately active (+++, ≥20 IC_50_ < 200 nM); less active (++, ≥200 IC_50_ < 2000 nM); inactive (+, IC_50_ ≥ 2000 nM). Activities of all compounds were estimated based on the best ranking pharmacophore model, Hypo1. The experimental and estimated activity values for the 20 training set compounds based on Hypo1 are listed in Table 2.

**Figure 5:**
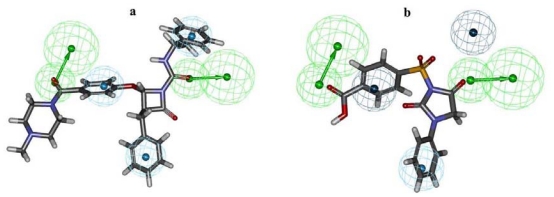
Mapping of most active compound **1** (**a**) and least active compound **20** (**b**) of training set over pharmacophore model Hypo1.

Analysis of the activity prediction of training set compounds revealed that all the most active compounds were predicted in the same scale, whereas only one moderately active compound was estimated as less active and three inactive compounds were estimated as less active compounds among the 20 compounds of training set. The estimated activity values of most and least active compounds of the training set based on Hypo1 were 0.27 and 4800 nM, respectively, which are very close to that of their experimental activity values (0.46 and 5900 nM). This result revealed that the structural characteristics which can explain the difference in their biological activities are present in Hypo1 (Figure 5a).

The most active compound **1** could map all the features of the best pharmacophore model, Hypo1, with a fit value of 9.04. The carbonyl oxygen atoms attached with the piperazine ring and azetidinone moiety were mapped onto the two HBA features. All three phenyl rings present in this most active compound mapped over three HY-AR features. The least active compound **20** in the training set maps Hypo1 with a fit value of 4.79 missed two HY-AR features as compared to compound **1**. Carbonyl group of imidazolidine-dione and the only carboxyl group of this least active compound mapped both the HBA features whereas the phenyl ring attached to the imidazolidine-dione mapped over one of the HY-AR features (Figure 5b).

### 2.2. Pharmacophore Validation

#### 2.2.1. Test Set Prediction Method

The validation of suggested pharmacohore model, Hypo1, was performed by two different validation methods, namely, test set prediction and Fischer randomization methods. A test set containing 97 compounds, representing diverse activity classes and different functional groups, is used in this validation process. These test compounds were imported into the DS and diverse conformers were built in the same manner as for training set compounds. The estimated activities of these test set compounds were calculated based on the geometric fit of these compounds over Hypo1. Analyses of the estimated activities of test set compounds demonstrated remarkable results. From the 97 test set compounds, 94 compounds showed error values less than 5 which is hardly different from the experimental and estimated activity values (Table 3).

**Figure 6:**
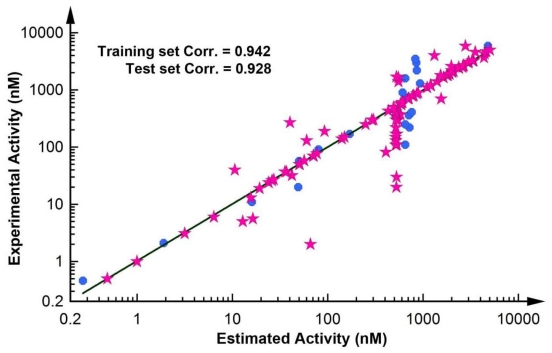
The correlation graph between experimental and estimated activity values based on Hypo1.

Eight out of nine of the most active compounds were estimated in the same activity scale, whereas the ninth compound was predicted as moderately active. Seventeen out of 26 moderately active compounds were estimated in the same scale, whereas the remaining nine were estimated as less active compounds. All the 40 less active compounds were estimated in the less active scale. Furthermore, only three of the 22 inactive compounds were predicted as less active compounds. Thus, the ability of Hypo1 to forecast the activity of test set compounds was very impressive and outstanding. A correlation value of 0.928 was achieved between experimental and estimated activities of test set compounds. A correlation plot showing the correlation between the experimental and estimated activity values of training and test set compounds was generated and displayed in Figure 6.

#### 2.2.2. Fischer Randomization Method

Another validation method based on Fischer randomization was also performed on the training set compounds to verify the quality of Hypo1. In this validation process, a confidence level of 95% was selected and thus 19 spreadsheets (Table 4) were generated.

The data obtained from this validation method did not produce any better statistical values compared with that of Hypo1. Out of the 19 runs, only three had a correlation value between 0.90 and 0.92 which was comparatively less than the correlation value of Hypo1. The total cost values of all randomized models and RMSD values were higher than Hypo1, which is not appropriate for a good pharmacophore model. Therefore, this validation test also endows the Hypo1 with a high level of assurance.

### 2.3. Search for New Potential Compounds Using Database Screening

The suggested pharmacophore model Hypo1 developed so far divulges a fairly accurate idea of the required molecular features for a new lead. Therefore, Hypo1 was applied as a search query to retrieve molecules with novel and desired attributes from chemical databases (Maybridge and Chembridge). A total of 2202 hit compounds, 1478 compounds from Maybridge and 724 compounds from Chembridge, respectively, were obtained. Molecular properties were calculated for all hit compounds retrieved from databases. The 181 hit compounds (124 from Maybridge and 57 from Chembridge database, respectively) with an estimated activity value closer to the most active compound in the training set were selected for further evaluation. These hits were further filtered by using Lipinsiki’s rule of five which evaluates drug-likeness, or determines if a chemical compound with a certain pharmacological or biological activity has properties that would make it feasible to be an orally active drug in humans. The 49 compounds of Chembridge database and 23 compounds from Maybridge database have satisfied the requirements of Lipinsiki’s rule of five for a drug-like compound. Thus, these 72 hit compounds that satisfied the Lipinsiki’s rule of five from a total of 181 hits were subjected to molecular docking.

### 2.4. Molecular Docking

**Figure 7:**
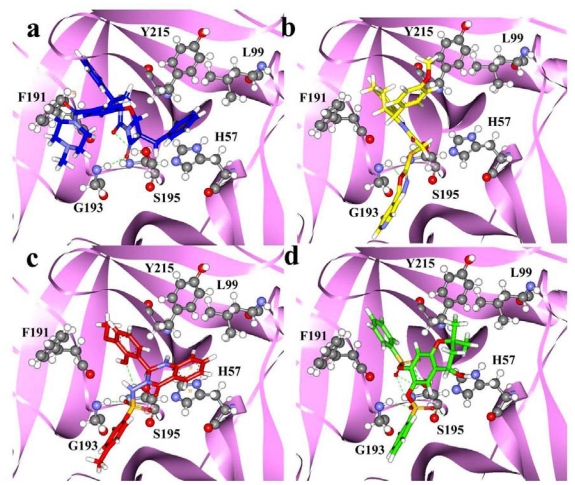
The molecular docking results: The binding modes and molecular interactions of compound **1** of training set (**a**); HTS12673 (**b**); BTB02076 (**c**); JFD00311 (**d**) at the binding site of chymase enzyme. The key active site residues and inhibitors are shown in stick and ball-stick forms, respectively. The hydrogen bonds between protein and inhibitors are shown in green dashed lines.

All of the 20 training set compounds along with the 72 database hits retrieved from the database screening process were docked into the protein active site using the *GOLD* (Genetic Optimization for Ligand Docking) docking program. *GOLD* fitness score which differentiates molecules on account of their interacting pattern is calculated for all molecules. The most active compound of training set (compound **1**) scored a docking score of 66.6 and exhibited various hydrogen-bonding interactions with the key active site residues (Figure 7a).

**Figure 8:**
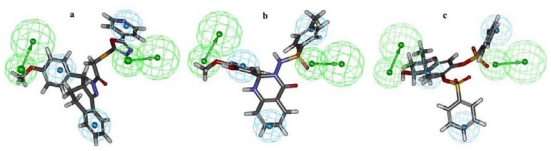
Pharmacophore mapping of three final hit compounds HTS12673 (**a**); BTB02076 (**b**) and JFD00311(**c**) over the selected pharmacophore model Hypo1.

**Figure 9:**
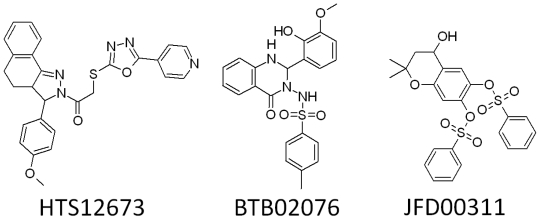
2D molecular structures of hit compounds HTS12673 (**a**), BTB02076 (**b**), and JFD00311(**c**).

Moreover, two of the carbonyl oxygen atoms near the middle ring that mapped on the HBA features of “Hypo1” showed hydrogen-bonding interactions with Gly193 and Ser195 residues of the active site. Previous studies of chymase have also divulged the importance of Gly193 and Ser195 as key amino acids in active site region of the enzyme [9,13]. Along with diverse hydrogen-bonding contacts, the phenyl group of compound **1**, which was mapped on the HY-AR feature of “Hypo1” showed π···σ interactions with the aromatic ring of residue F191. Moreover, compound **1** also showed hydrophobic interactions with Y215 and L99 amino acids. Several hit compounds obtained from database screening process also showed high *GOLD* fitness scores and formed interactions with the active site residues. The hit compounds that showed a fitness score of more than 66 were selected as final hits for further evaluation process. Intriguingly, all the final three compounds were obtained from Maybridge database and none from the Chembridge database. Compound HTS12673 which showed an estimated activity value of 6.716 nM has scored a *GOLD* fitness score of 78.73. It has also exhibited key interactions with the important amino acids like Gly193, Ser195, Y215, and H57 at the active site of the enzyme (Figure 7b). The phenyl part of anisole ring and pyridine ring that mapped over the HY-AR features of “Hypo1” instigated the improved binding of this compound through better hydrophobic interactions. Compound BTB02076, which was also retrieved from the Maybridge database, with an estimated activity value of 8.605 nM has shown a *GOLD* fitness score of 72.40. This compound has formed various close contacts that lead to the important ligand-enzyme interaction such as hydrogen bonding interactions with Gly193, Ser195 and hydrophobic interactions with Phe191 amino acid in the active site of the enzyme (Figure 7c). Moreover, important π···π interactions between the fused ring system of BTB02076 and the side chain imidazole ring of His57 amino acid were also revealed. Furthermore, it also showed hydrophobic interactions with Y125 and L99 amino acid residues of protein through the hydrophobic groups mapped over HY-AR features of Hypo1. Third hit, JFD00311, with the estimated activity value of 4.661 nM and *GOLD* fitness score of 74.51 has formed hydrogen bond network with the active site residues Gly193, and Ser195 (Figure 7d). The benzene rings and oxygen atoms of the benzenesulfonic acid moieties in this hit compound that overlaid the HY-AR and HBA features of “Hypo1”, respectively, enabled considerable hydrophobic and polar interactions with the important amino acids in the active site. The mapping of these top three database final hits on Hypo1 and their 2D molecular structures are depicted in Figures 8 and 9, respectively.

All three hit compounds have mapped the entire features of the best pharmacophore model, Hypo1. Thus, in the design of potent inhibitors of chymase, compounds HTS12673, BTB02076, and JFD00311 which showed important results with respect to all properties such as estimated activity, calculated drug-like properties and better *GOLD* fitness scores can be proposed as potential leads. Novelty search using *SciFinder Scholar* and *PubChem compound* search has also ascertained that these hits were not reported earlier for chymase inhibition.

### 2.5. Density Functional Theory Calculations

#### 2.5.1. Analysis of Orbital Energies

The electrostatic features impacting the inhibitory effect of chymase inhibitors have been investigated aiming at providing useful information for understanding the structure inhibition relationships of chymase inhibitors. Structures of the most and least active compounds of the training set are optimized along with the three final database hit compounds at B3LYP/6-31G* level. Statistically significant factors such as HOMO, LUMO, and MESP, for all compounds are calculated. According to Fukui’s frontier orbital approximation, the frontier orbitals HOMO and LUMO of a chemical species are very important in defining its reactivity. Fukui first recognized the importance of frontier orbitals as principal factors governing the ease of chemical reactions and the stereoselective path while Parr and Yang demonstrated that most frontier theories can be rationalized from DFT.

**Figure 10:**
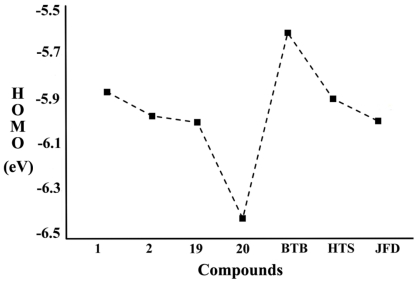
HOMO energies (eV) of chymase inhibitors along with potent hits.

When the whole dataset of molecules was taken into account, an apparent trend of inhibitory activity (IC_50_) data with an increase in HOMO energy was observed (Figure 10).

For all compounds, HOMO energy ranges between −5.619 and −6.415 eV. High value of *E*_HOMO_ is likely to indicate a tendency of the molecule to donate electrons to appropriate acceptor molecule of low empty molecular orbital energy. The correlation of HOMO energies with IC_50_ data indicates that the HOMO of the inhibitor may transfer its electrons to less energy, LUMO, of some amino residues in the active site of chymase. The calculations show that compounds **1** and **20** have shown the highest (−5.873 eV) and lowest (−6.415 eV) HOMO level energies respectively. This trend is in good agreement with the experimental observations suggesting that compounds **1** and **20** have exhibited the highest (0.46 nM) and lowest (5900 nM) inhibitory profile, respectively, in all investigated chymase inhibitors. While BTB (BTB02076) has shown highest (−5.619 eV) HOMO level energy among hit compounds even higher than HOMO energy level of compound **1**, the other two hit compounds also showed higher *E*_HOMO_ than the least active compound of the data set. In a previous study, a high HOMO energy level also played an important part in activity of the most active dual and selective LOX inhibitors [61]. Moreover, a clear trend between the inhibitory activity (IC_50_) data and LUMO energy of all compounds was also revealed. For all compounds, LUMO energy ranged between −0.631 and −2.275 eV. Compound **1** and BTB showed highest LUMO level energies; and least active compounds 19 and 20 demonstrated LUMO with lowest energies.

**Figure 11:**
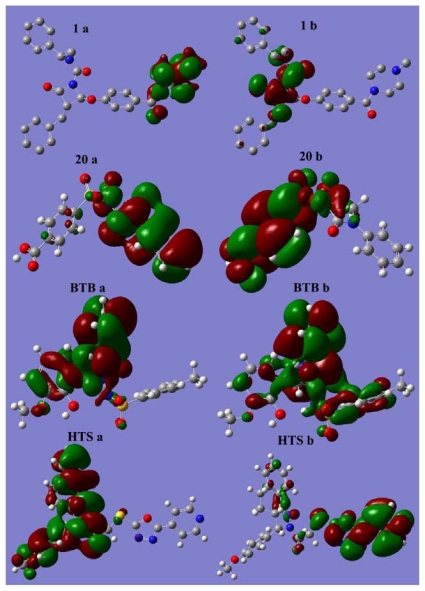
Plots of HOMO (**a**) and LUMO (**b**) of most active (**1**), and least active (**20**) compounds along with potent hits BTB (BTB02076) and HTS (HTS12673).

HOMO and LUMO sites are plotted onto the molecular surface of most active (**1**) and least active (**20**) compounds of the data set along with the two hit (BTB, HTS) compounds (Figure 11).

Most often, the heteroaromatic rings, which contain the heteroatoms such as nitrogen and oxygen, are the regions in all these compounds that can act as electron donors or acceptors to the active site of the chymase. Experimental study also deduced that introduction of heteroatoms to the inhibitor compound enhanced its stability in human plasma (**20**). For instance, the placement of an ethoxy group in compound **2** instigated its stability. Electron donor rings can be identified as those with the greatest electron density from the HOMO. In the case of compound **1**, HOMO is scattered over the 4-methylpiperazine moiety together with the carbonyl group and LUMO is spread over the region 2-hydroxyl-4-oxoazetidine containing heteroatoms like oxygen and nitrogen. Docking results also showed that this region of compound **1** is involved in important interactions with the key residues of protein. For compound **20**, HOMO is composed of aniline ring and LUMO spreads over sulfonyl and benzoic acid groups. LUMO plot over methylbenzenesulfonamide group in hit compounds BTB showed hydrogen bonding interactions with important amino acids Gly193 and Ser195 at the active site of the enzyme. Whereas the HOMO plot is scattered on 2-methoxyphenol group and dihydroquinazolin moiety, the six membered ring part of dihydroquinazolin group is involved in important π···π interactions with the side chain imidazole ring of His57 amino acid. For HTS hit compound, HOMO and LUMO are composed of methoxybenzene, benzoindazole moieties, and oxadiazole substituted pyridine moiety, respectively. Overlay of HTS on “Hypo1” and its docking with the protein also speculated the involvement of these groups in key interactions with the active site of protein. The effect of the orbital energies on the inhibition activities can be associated with the charge transfer, π···π, or π···σ stacking between inhibitors and aromatic amino acid residues in the binding site of chymase. The result of molecular docking studies on chymase inhibitors also proved the presence of such kind of interactions.

#### 2.5.2. Molecular Electrostatic Potential (MESP) Profiles

**Figure 12:**
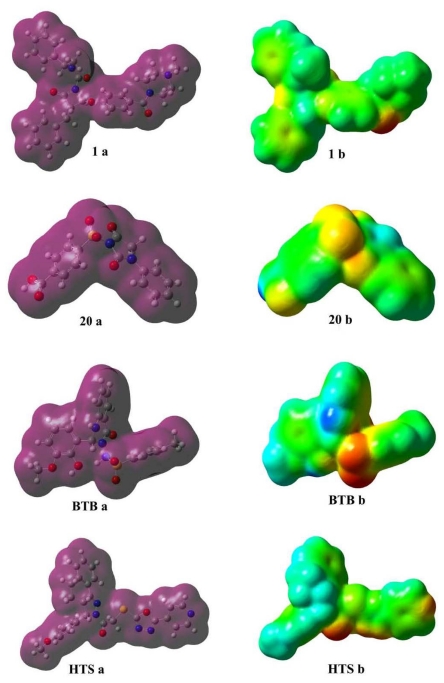
Differential maps of total density (**a**) and MESP (**b**) of most active, **1**, and least active, **20**, compounds along with potent hits BTB (BTB02076) and HTS (HTS12673). The red and the blue color represent the electronegative and electropositive potentials whereas the green represents a potential halfway between the two extremes.

Electrostatic potential is widely used in characterizing molecules, especially for biomolecules, and takes special effect in the biomolecular recognition and in the prediction of the functional sites [62]. Nam *et al*. reported their discovery that electrostatic interactions accounted for the majority of the rate acceleration in the mechanism of RNA transphosphorylation in solutions catalyzed by the hairpin ribozyme [63]. Daga and Doerksen have stated the binding mode and the role of stereoelectronic properties in binding of spiroquinazolinones showing phosphodiesterase 7 (PDE7) inhibitory activities [64]. Recently, the electrostatic funnel illuminated from three-dimensional mapping of the electrostatic potential was reported by Dehez *et al*., driving the diphosphate nucleotide rapidly toward the bottom of the internal cavity of membrane-protein mitochondrial ADP/ATP carrier by forming a privileged passageway [65]. Considering these discoveries comprehensively, we supposed that the electrostatic potential of the inhibitor also played a significant role in the binding and interaction with chymase together with orbital energy and consequently influenced the inhibition effect. The 3D isosurface maps of MESP were interpolated on the electron density surfaces of constant electron charge density (0.0004 e/au^3^). As is well known, the electrostatic potential is defined as the interacted energy of a positively remote charge point with the nuclei and the electrons of a molecule. The 3D plots of electron density (ED) and the MESP for compounds **1**, **20**, BTB and HTS are shown in Figure 12.

The coloring area of the surface represents the overall molecular charge distribution with the electrostatic potential. As for the compounds in this study, the electronegative potential (MESP_min_) was coded with red on the MESP maps in a range from 202.16 to 152.27 kcal/mol indicating a strongest attraction while the interpolated blue map represents the electropositive potential (MESP_max_) of a strongest repulsion varying from 15.68 to 42.67 kcal/mol. The predominance of green region in the MESP surfaces corresponds to a potential halfway between the two extremes that are indicated in red and blue colors, respectively.

MESP plotted onto constant electron density surface for most active compound **1** showed the most electronegative potential region (red color) over the oxygen atom of the carbonyl group near the piperazine moiety. However, in the case of the least active compound **20**, most negative potentials due to sulfonyl and carbonyl oxygen atoms are missing. For hit compounds, appearance of localized negative potential regions located at the oxygen atoms of the carbonyl and sulfonyl groups and nitrogen of the pyridine ring are consistent with the docking results which recognized this region as hydrogen bond acceptor. Moreover, one more prominent localized negative charged region protruding over the oxadiazole group was oriented adjacent to Gly193, to be recognized as a hydrogen bond acceptor. The strong electrostatic interaction of the negative potential with key residues Gly193 and Ser195, namely the formation of the hydrogen bond, will enhance the inhibition effect substantially together with the orbital interaction through the exchange of energy. The blue electropositive maps of these compounds were mainly distributed over the methyl group. The hydrogen atoms attached to the six-membered rings also bear the maximum brunt of positive charge (blue region). Due to the accumulation of positive potential, these moieties exhibited π···π and π···σ interactions with the aromatic residues of active site. These molecular electrostatic potential features are also in concert with the key chemical features (HBA and HY-AR) of pharmacophore model (Hypo1) which was successfully employed as a 3D structural query for virtual screening of databases for the identification of new potent chymase inhibitors. Thus electrostatic potential of the inhibitors can play a significant role in the binding and interaction with chymase together with orbital energies, and consequently influence the inhibition effect.

## 3. Materials and Methods

### 3.1. Pharmacophore Modeling

#### 3.1.1. Selection of Training Set Compounds and Diverse Conformation Generation

A set of 117 structurally distinct compounds reported as chymase inhibitors with their diverse experimentally known inhibitory activity (IC_50_) data was compiled from the literature such as life science journals [14–20,55,66–68]. All of the inhibitory activities were obtained using the same biological assay method [14]. To form a training set, 20 compounds with distinctive structural motif and wide activity range (0.46 to 5900 nM) were selected. For all compounds in the training set, energy minimization process was performed with *CHARMM* forcefield. Poling algorithm was applied to generate a maximum of 255 diverse conformations with the energy threshold of 20 kcal·mol^−1^ above the calculated energy minimum for every compound in the dataset. These conformers were generated using *Diverse Conformer Generation* protocol running with *Best*/*Flexible* conformer generation option as available in Accelrys Discovery Studio v2.5 (DS), Accelrys, San Diego, CA, USA. This method ensures the best coverage of conformational space by performing a more rigorous energy minimization in both torsional and cartesian space by using poling algorithm.

#### 3.1.2. Pharmacophore Model Generation

All the 20 training set compounds associated with their conformations were submitted to the *HypoGen* module of DS. The *HypoGen* algorithm implemented for the pharmacophore hypothesis generation process is executed in three phases, namely, constructive, subtractive, and optimization phases. In constructive phase, identification of features common to the most active compounds takes place whereas all pharmacophoric features that are also present in the least active compounds are removed in subtractive phase. Finally, in the optimization phase, the hypothesis score is improved by regression parameters which are used for the estimation of the activity value of each training set compound. The relationship between the geometric fit value and activity value is utilized for this computation. Pharmacophore hypotheses showing best correlation in the 3D arrangement of features in a given training set compounds with the corresponding pharmacological activities are formed and ranked. Several structure activity relationship (SAR) pharmacophore models were derived from training set compounds using *HypoGen* module of DS. In this study, the top 10 hypotheses which were returned by the hypotheses generation process with significant statistical parameters were selected for further calculations.

#### 3.1.3. Pharmacophore Model Validation and Database Searching

The generated quantitative pharmacophore model was validated to find out whether it is competent enough to identify the active structures and estimate their activity values precisely. This validation process was performed based on test set prediction and Fischer randomization methods. In developing statistical models, the following three cross-validation methods are often used to examine a model or predictor for its effectiveness in practical application: independent dataset test, subsampling test, and jackknife test [69]. However, of the three test methods, the jackknife test is deemed the most objective [29]. The reasons are as follows. (i) For the independent dataset test, although all the samples used to test the model or predictor are outside the training dataset used to train it so as to exclude the “memory” effect or bias, the way of how to select the independent samples to test the model or predictor could be quite arbitrary unless the number of independent proteins is sufficiently large. This kind of arbitrariness might result in completely different conclusions. For instance, a model or predictor achieving a higher success rate than the other model or predictor for a given independent testing dataset might fail to keep so when tested by another independent testing dataset [69]; (ii) For the subsampling test, the concrete procedure usually used in literatures is the 5-fold, 7-fold or 10-fold cross-validation. The problem with this kind of subsampling test is that the number of possible selections in dividing a benchmark dataset is an astronomical figure even for a very simple dataset, as elucidated demonstrated by Equations 28–30 in [70]. Therefore, in any actual subsampling cross-validation tests, only an extremely small fraction of the possible selections are taken into account. Since different selections will always lead to different results even for a same benchmark dataset and a same model or predictor, the subsampling test cannot avoid the arbitrariness either. A test method unable to yield a unique outcome cannot be deemed as a good one; (iii) In the jackknife test, all the samples in the benchmark dataset will be singled out one-by-one and tested by the model or predictor trained by the remaining samples. During the process of jackknifing, both the training dataset and testing dataset are actually open, and each sample will be in turn moved between the two. The jackknife test can exclude the “memory” effect. Also, the arbitrariness problem as mentioned above for the independent dataset test and subsampling test can be avoided because the outcome obtained by the jackknife cross-validation is always unique for a given benchmark dataset. Accordingly, the jackknife test has been increasingly and widely used by those investigators with strong math background to examine the quality of various predictors (see e.g., [30,71–75]). However, to reduce the computational time, we adopted the independent testing dataset cross-validation in this study as done by many investigators with SVM as the prediction engine. A test set comprising 97 compounds with experimentally known chymase inhibitory activity values was used in test set prediction method. *Ligand Pharmacophore Mapping* protocol running with *BEST*/*Flexible* conformation generation option was used to map the test set compounds. Fischer randomization method as available in DS was applied on training set compounds to prove that the generated pharmacophore model was not obtained by chance. A pharmacophore is only useful as a predictive model in finding novel, potential leads suitable for further development only if it is able to detect the compounds with known inhibitory activity. In order to identify new potential lead compounds, the selected pharmacophore model was subsequently used as 3D structural search query to screen the Maybridge and Chembridge chemical databases consisting of 60,000 and 50,000 of structurally assorted compounds, respectively. All queries were performed using *Ligand Pharmacophore Mapping* protocol running with *Best*/*Flexible search* method in DS. To be retrieved as a hit, a molecule must fit all the features of the pharmacophore hypothesis. The hits obtained through database screening were further filtered using Lipinsiki’s rule of five in order to carry only drug-like compounds in further studies.

### 3.2. Molecular Docking

Computational docking operation is a useful vehicle for investigating the interaction of a protein receptor with its ligand and revealing their binding mechanism as demonstrated by a series of studies [18,46,76–84]. Docking plays a significant role in predicting binding orientation and affinity of small molecule drug candidates to their protein targets with known 3D structures [85,86]. Hence, docking serves as an important tool in the rational computer-assisted drug design [87,88]. *GOLD* 4.1 (Genetic Optimization for Ligand Docking) from Cambridge Crystallographic Data center, UK uses a genetic algorithm for docking ligands into protein binding sites to explore the full range of ligand conformational flexibility with partial flexibility of protein [89]. In this study, it has been utilized for the docking of training set compounds along with the new hits retrieved from chemical databases. Protein coordinates from the crystal structure of chymase co-crystallized with β-ketophosphonate (PDB ID: 1T31), determined at a resolution of 1.9 Å were used to define the active site [9]. All the water molecules present in the protein were removed and hydrogen atoms were added. The active site was defined with a 10 Å radius around the ligand present in the crystal structure. At the end of the computation, the 10 top-scoring conformations of every ligand were saved. Early termination option was applied to pass over the genetic optimization calculation when any five conformations of a particular compound were envisaged within an RMS deviation value of 1.5 Å. The *GOLD* fitness score is calculated from the contributions of hydrogen bond and van der Waals interactions between the protein and ligand, intramolecular hydrogen bonds and strains of the ligand. The protein–ligand interactions were scrutinized by DS.

### 3.3. Density Functional Theory (DFT) Calculations

As far as computational technique is considered, many practices have ascertained that DFT, which takes into account the exchange and correlation effects effectively, is most likely one of the best methods to study medium-size or larger molecular systems and appropriate for QSAR study, with exhibiting excellent performance than semiempirical method or some other *ab initio* methods. Complete geometry optimization for data set compounds was carried out using DFT with Becke’s three-parameter exchange potential and the Lee-Yang-Parr correlation functional (B3LYP), using basis set 6-31G* level [90]. A useful kind of net atomic charges, called electrostatic potential (ESP)-fitting charges, were derived from the DFT calculated molecular electrostatic potential distribution using CHelpG method, which produces charges fit to the electrostatic potential at points selected. Vibrational frequencies were computed at the same B3LYP/6-31G* level to characterize the stationary points on the corresponding potential energy surfaces. All calculations were performed using the Gaussian 03 suite of programs.

#### 3.3.1. Data Set of DFT Study

Based on structural diversity and wide biological activity range, four chymase inhibitors including most and least active compounds, were selected from the training set. While, three final hits BTB02076, HTS12673, JFD00311 retrieved from Maybridge database by the selected pharmacophore model, which showed important results with respect to all properties like molecular interactions with the active site components, estimated activity, calculated drug-like properties, and high *GOLD* fitness score, were also selected. Thus, data set employed for DFT study consisted of seven compounds. Various quantum-chemical descriptors such as LUMO, HOMO, and locations of molecular electrostatic potentials (MESP), were computed.

#### 3.3.2. Calculation of Molecular Electrostatic Potential (MESP)

The mapping of the electrostatic potential is an established technique for investigation of biologically active compounds because it plays a key role in the initial steps of ligand-receptor interactions. The formatted checkpoint files of the compounds generated by the geometric optimization computation were used as input for *CUBEGEN* program interfaced with *Gaussian 03* program to compute the MESP. The MESP isopotential surfaces was produced and superimposed onto the total electron density surface (0.0004 e/au^3^). The electrostatic potential of the whole molecule is finally obtained by superimposing the electrostatic potentials upon the total electron density surface of the compound.

## 4. Conclusion

Since user-friendly and publicly accessible web-servers represent the future direction for developing practically more useful models or predictors [91], we shall make efforts in our future work to provide a web-server for the method presented in this study.

Combining various theoretical methods like pharmacophore modeling, database screening, molecular docking and DFT calculations, an investigation for identification of novel chymase inhibitors and to specify the key factors crucial for the binding and interaction between chymase and inhibitors was performed. The highly correlating (*r* = 0.942) pharmacophore model (Hypo1) with two hydrogen bond acceptors, and three hydrophobic aromatic features was generated. After successfully validating “Hypo1” using test set and Fischer randomization methods, it was further used in database screening. Hit compounds were subjected to various drug-like filtrations and molecular docking studies. Finally, three structurally diverse compounds with high estimated activity and strong molecular interactions with key active site amino acids were identified. Furthermore, a DFT study, which articulated the influence of the electrostatic features of compounds on their inhibitory activity well, was performed. Analysis of orbital energies and plots of MESP has shown very clear trends between electronic properties and inhibitory activity (IC_50_) data. An increasing trend was observed between IC_50_ and HOMO energy values. The molecular electrostatic potential features were also consistent with the key chemical features of “Hypo1” thus successfully validating “Hypo1” by the DFT method. Therefore, the results of this study will be helpful, not only in the development of new potent hits for chymase, but also in providing a better understanding of the interaction between the chymase and inhibitors. This will in turn assist in the rational design of novel potent enzyme inhibitors.

## Figures and Tables

**Table 1:** Statistical details for top 10 scoring hypotheses.

Hypothesis	Total cost	ΔCost a	RMSD (Å)	Correlation (*r*)	Features
1	89.663	92.703	1.176	0.942	HBA, HBA, HY-AR, HY-AR, HY-AR
2	91.454	90.912	1.25	0.934	HBA, HBA, HY-AR, HY-AR, HY-AR
3	94.811	87.555	1.348	0.924	HBA, HY-AR, HY-AR, RA
4	95.086	87.28	1.388	0.919	HBA, HBA, HY-AR, HY-AR, HY-AR
5	95.379	86.987	1.387	0.919	HBA, HY-AR, RA, RA
6	95.458	86.908	1.396	0.918	HBA, HBA, HY-AR, HY-AR, HY-AR
7	95.656	86.71	1.406	0.916	HBA, HBA, HY-AR, HY-AR, HY-AR
8	95.855	86.511	1.409	0.916	HBA, HBA, HY-AR, HY-AR, HY-AR
9	95.538	86.828	1.411	0.916	HBD, HY-AR, RA, RA
10	96.124	86.242	1.421	0.915	HBA, HBA, HY-AR, HY-AR, HY-AR

Null cost = 182.366, fixed cost = 75.791, configuration cost = 16.606; all cost values are in bits;

a ΔCost = Null cost − Total cost; Abbreviations used for features: HBA, Hydrogen-bond acceptor; HY-AR, hydrophobic aromatic; RA, ring aromatic; HBD, Hydrogen-bond donor.

**Table 2:** Experimental biological activity data and estimated IC_50_ values of training set compounds based on pharmacophore model Hypo1.

Compound	Experimental activity (nM)	Estimated activity	Error	Activity scale a	Estimated activity scale
1	0.46	0.27	−1.7	++++	++++
2	2.1	1.9	−1.1	++++	++++
3	11	16	1.4	++++	++++
4	20	49	2.5	+++	+++
5	57	50	−1.1	+++	+++
6	91	80	−1.1	+++	+++
7	110	650	5.9	+++	++
8	170	170	1	+++	+++
9	220	720	3.3	++	++
10	250	650	2.6	++	++
11	360	710	2	++	++
12	410	760	1.9	++	++
13	730	660	−1.1	++	++
14	900	610	−1.5	++	++
15	1300	930	−1.4	++	++
16	1600	650	−2.6	++	++
17	2200	860	−2.6	+	++
18	3000	850	−3.5	+	++
19	3500	830	−4.2	+	++
20	5900	4800	−1.2	+	+

a Activity scale: most active (++++, IC_50_ < 20 nM); moderately active (+++, ≥20 IC_50_ < 200 nM); less active (++, ≥200 IC_50_ < 2000 nM); inactive (+, IC_50_ > 2000 nM).

**Table 3:** Test set compounds listed with their experimental, estimated activities and error values.

Name	IC50 nM		Error c	Activity scale d		Name	IC50 nM		Error	Activity scale	
	Exp. a	Est. b		Exp.	Est.		Exp.	Est.		Exp.	Est.
21	0.5	0.48	−1.0	++++	++++	70	500	491.7	−1.0	++	++
22	1	1.00	−1.0	++++	++++	71	550	592.4	1.0	++	++
23	2	65.84	32.9	++++	+++	72	580	579.668	−1.0	++	++
24	3.1	3.16	1.0	++++	++++	73	600	601.005	1.0	++	++
25	5	12.84	2.5	++++	++++	74	609	609.043	1.0	++	++
26	5.6	16.38	2.9	++++	++++	75	700	1545.30	2.2	++	++
27	6	6.38	1.0	++++	++++	76	700	697.894	−1.0	++	++
28	13	15.57	1.1	++++	++++	77	710	709.97	−1.0	++	++
29	19	19.30	1.0	++++	++++	78	780	779.896	−1.0	++	++
30	20	524.55	26.2	+++	++	79	800	798.863	−1.0	++	++
31	24	23.99	−1.0	+++	+++	80	860	860.59	1.0	++	++
32	26	26.03	1.0	+++	+++	81	890	889.222	−1.0	++	++
33	27	27.18	1.0	+++	+++	82	890	889.127	−1.0	++	++
34	30	526.07	17.5	+++	++	83	1100	1103.48	1.0	++	++
35	32	42.12	1.3	+++	+++	84	1200	1209.95	1.0	++	++
36	37	37.00	−1.0	+++	+++	85	1200	1193.16	−1.0	++	++
37	37	35.56	−1.0	+++	+++	86	1400	1399.23	−1.0	++	++
38	40	10.60	−3.7	+++	+++	87	1400	552.461	−2.5	++	++
39	50	50.85	1.0	+++	+++	88	1650	1645.71	−1.0	++	++
40	50	50.17	1.0	+++	+++	89	1650	551.849	−2.9	++	++
41	58	56.72	−1.0	+++	+++	90	1700	524.94	−3.2	++	++
42	70	71.11	1.0	+++	+++	91	1800	1821.04	1.0	++	++
43	70	70.25	1.0	+++	+++	92	1800	1776.13	−1.0	++	++
44	77	76.65	−1.0	+++	+++	93	1800	1530.73	−1.1	++	++
45	82	408.48	4.9	+++	++	94	1900	1901.07	1.0	++	++
46	109	517.17	4.7	+++	++	95	1900	1876.01	−1.0	++	++
47	110	532.07	4.8	+++	++	96	2040	2022.46	−1.0	+	+
48	130	518.66	3.9	+++	++	97	2100	2095.67	−1.0	+	+
49	130	60.13	−2.1	+++	+++	98	2200	1991.66	−1.1	+	++
50	140	140.46	1.0	+++	+++	99	2400	2386.02	−1.0	+	+
51	150	514.80	3.4	+++	++	100	2500	2594.48	1.0	+	+
52	150	149.81	−1.0	+++	+++	101	2500	2373.56	−1.0	+	+
53	170	531.86	3.1	+++	++	102	2600	2653.51	1.0	+	+
54	170	520.35	3.0	+++	++	103	2600	1980.42	−1.3	+	++
55	190	92.38	−2.0	+++	+++	104	2700	2668.93	−1.0	+	+
56	220	519.50	2.3	++	++	105	3000	2980.04	−1.0	+	+
57	250	249.79	−1.0	++	++	106	3100	3021.26	−1.0	+	+
58	270	40.16	−6.7	++	++	107	3200	3351.54	1.0	+	+
59	300	546.21	1.8	++	++	108	3300	3370.61	1.0	+	+
60	300	521.04	1.7	++	++	109	3300	3333.56	1.0	+	+
61	300	301.67	1.0	++	++	110	3300	3277.62	−1.0	+	+
62	300	289.88	−1.0	++	++	111	3700	4354.53	1.1	+	+
63	370	580.52	1.5	++	++	112	4000	1316.33	−3.0	+	++
64	380	531.50	1.3	++	++	113	4300	4440.06	1.0	+	+
65	400	517.75	1.2	++	++	114	4600	3529.94	−1.3	+	+
66	400	513.35	1.2	++	++	115	4700	4603.40	−1.0	+	+
67	430	515.92	1.2	++	++	116	5000	5036.50	1.0	+	+
68	430	515.00	1.1	++	++	117	5860	2779.37	−2.1	+	+
69	430	431.38	1.0	++	++						

a Exp.: Experimental activity;

b Est.: Estimated activity;

c Value in the error column represents the ratio of the estimated activity to the experimental activity or its negative inverse if the ratio is less than one;

d Activity scale: most active (++++, IC_50_ < 20 nM); moderately active (+++, ≥20 IC_50_ < 200 nM); less active (++, ≥200 IC_50_ < 2000 nM); inactive (+, IC_50_ > 2000 nM).

**Table 4:** Results of cross-validation by Fischer randomization using DS.

Trial No.	Total cost	Fixed cost	RMSD	Correlation (*r*)
Hypo1	89.663	75.791	1.176	0.942
Results after randomization				
1	114.486	77.911	1.821	0.858
2	108.259	72.031	1.796	0.863
3	98.26	74.85	1.529	0.9
4	113.25	77.605	1.851	0.851
5	112.27	77.909	1.729	0.874
6	108.84	75.77	1.749	0.869
7	141.304	78.861	2.463	0.717
8	109.86	72	1.857	0.852
9	112.265	77.915	1.849	0.851
10	113.941	77.584	1.774	0.867
11	101.143	72.068	1.593	0.894
12	116.666	74.077	1.959	0.834
13	114.356	69.48	1.97	0.834
14	98.277	77.638	1.433	0.913
15	108.878	72.047	1.753	0.872
16	117.228	78.041	1.961	0.831
17	102.183	78.085	1.359	0.926
18	113.597	77.563	1.891	0.843
19	106.121	74.044	1.706	0.876
